# Comparative Genomic Analysis of Two Bat Poxviruses in the Genus *Vespertilionpoxvirus*

**DOI:** 10.3390/v18070706

**Published:** 2026-06-26

**Authors:** Chi Zhang, Kyle Heye, Davide Lelli, Loubna Tazi, Stefan Rothenburg

**Affiliations:** 1 Department of Medical Microbiology and Immunology, School of Medicine, University of California Davis, Davis, CA 95616, USA; chizhang@ucdavis.edu (C.Z.); krheye@ucdavis.edu (K.H.); ltazi@health.ucdavis.edu (L.T.); 2 Istituto Zooprofilattico Sperimentale della Lombardia e dell’Emilia Romagna (IZSLER), Via Bianchi 9, 25124 Brescia, Italy; davide.lelli@izsler.it

**Keywords:** poxvirus, eptesipox virus, hypsugopox virus, host range factor, bat viruses

## Abstract

Poxviruses are large double-stranded DNA (dsDNA) viruses that cause important human and animal diseases, including smallpox and mpox. Poxviruses have also been identified in diverse bat populations; however, their potential for zoonotic transmission and adaptation to other mammalian hosts remains poorly understood. Poxviruses encode numerous immunomodulatory proteins that contribute to virulence, immune evasion, and host range. In this study, we performed a comparative genomic analysis of two bat-associated poxviruses belonging to the genus *Vespertilionpoxvirus*: hypsugopox virus (HYPV) and eptesipox virus (EPTV). Our analyses revealed 24 novel putative ORFs in HYPV and three in EPTV, thereby substantially expanding the inferred coding capacity of these viruses. Comparative analyses further revealed gene duplication and fragmentation events affecting several virulence and host range factors, as well as other unusual genomic features, including the presence of two divergent E3L homologs in EPTV. Together, our findings provide new insights into the genome evolution and potential host adaptation of bat-associated poxviruses and establish a foundation for future functional studies of *Vespertilionpoxvirus* biology, host–virus interactions, and zoonotic potential.

## 1. Introduction

Bats are known or suspected reservoirs of many important zoonotic viruses, including severe acute respiratory syndrome coronavirus (SARS-CoV), severe acute respiratory syndrome coronavirus 2 (SARS-CoV-2), and Middle East respiratory syndrome coronavirus (MERS-CoV), as well as Ebola virus (EBOV), Marburg virus (MARV), Nipah virus (NiV), and Hendra virus (HeV). These viruses can cause severe diseases with high fatality rates in humans [1,2,3,4,5,6]. Increased interest in bat-borne viruses has led to the identification of many previously unknown viruses in recent years, including poxviruses [7,8,9,10,11].

Poxviruses are large double-stranded DNA viruses that replicate exclusively in the cytoplasm of cells. The family *Poxviridae* comprises two subfamilies: *Entomopoxvirinae*, which infect insects, and *Chordopoxvirinae*, which infect vertebrates and currently include 18 recognized genera [12]. They contain several important human pathogens, including variola virus (VARV), the causative agent of smallpox, monkeypox virus (MPXV), the causative agent of mpox, vaccinia virus (VACV), which is used as a smallpox and mpox vaccine, and cowpox viruses (CPXV) [13]. In addition, many poxviruses are important animal pathogens, including myxoma virus (MYXV), which has been instrumental in shaping our understanding of virus–host co-evolution and host switches [14].

Poxvirus genomes range from approximately 122 to 460 kb and contain densely packed genes. Genes essential for replication and virion formation are highly conserved and located near the center of the genome, whereas genes involved in host interactions and virulence are more frequently located towards the termini [15]. The two termini of the poxvirus genome are covalently closed and contain inverted terminal repeats (ITRs) with identical sequences in inverted orientation. Poxvirus genomes are highly adaptable. Common mechanisms of poxvirus evolution include point mutations, gene loss, gene duplication and consequent neofunctionalization, expansion, and contraction of their ITR regions, which often lead to differences in gene copy number, homologous and non-homologous recombination, and horizontal gene transfer from their hosts or other microorganisms [16,17,18].

Poxvirus entry into host cells is generally mediated by highly conserved cellular surface molecules rather than species-specific receptors, and successful replication largely depends on post-entry interactions with the host antiviral immune defenses [19,20]. The host range of poxviruses varies greatly, from viruses that productively infect only a single species, such as VARV (human-specific), to others, sometimes closely related viruses, such as CPXVs, which have naturally caused disease in more than 60 different host species [21]. Although the precise mechanisms determining poxvirus host range are not fully understood, a group of genes has been described that influences host and cell line tropism, collectively referred to as host range genes [22,23,24]. In orthopoxviruses, the number of host range genes roughly correlates with the size of their host range [24]. In addition, sequence variations in poxvirus host range genes have been shown to be important to virus replication and host tropism [23].

Although several bat poxvirus species have been recently identified, their host ranges and potential for spillover into other mammals remain largely unknown [7,8,9]. The recent report of a laboratory-confirmed human infection with Israeli *Rousettus aegyptiacus* poxvirus highlights the importance of investigating bat poxviruses and evaluating their zoonotic potential [25]. Comparative genomic analyses on conserved and unique genes can provide important insights into the putative virulence and host-range determinants of these viruses. However, genomic information on bat poxviruses remains limited, as only three bat poxviruses have had their nearly complete genomes sequenced [8,9,26]. Among these are eptesipox virus (EPTV) and hypsugopox virus (HYPV), the only known members of the genus *Vespertilionpoxvirus*, which are found in a sister clade (clade II poxviruses) to *Orthopoxvirus* and *Centapoxvirus* genera. EPTV was initially identified from the wings and joints of several sick big brown bats (*Eptesicus fuscus*) with progressive joint swelling and inability to fly in Washington, USA [7]. Its genome was subsequently sequenced and found to be 176,688 nucleotides in length and to contain 191 open reading frames (ORFs), 11 of which lacked homologs in other known poxviruses [26]. EPTV was later isolated from lesions in infected big brown bats in Saskatchewan, Canada, which shared 99.7% sequence identity with the Washington isolate [27]. HYPV was detected in deceased insectivorous bats (*Hypsugo savii*) during viral surveillance programs in Italy. Necropsy showed lymphoplasmacytic pneumonia in two bats, while no apparent pathological lesions were observed in another infected bat [9,28]. A total of 161 ORFs were annotated in the 166,600 bp HYPV genome, while the precise extent of the ITR region was not resolved [9]. However, because the original annotation relied on sequence similarity to EPTV, potentially divergent ORFs may have been overlooked. The incomplete annotation may lead to inaccurate conclusions regarding *Vespertilionpoxvirus* gene content and genome organization.

In this study, we reannotated the HYPV genome and performed a comparative genomic analysis of the two vespertilionpoxviruses. We identified multiple previously unannotated ORFs in HYPV and three small ORFs in EPTV, characterized gene deletions, fragmentations, and duplication events. These findings provide a more comprehensive view of the genomic architecture of the two bat poxviruses, which offer a foundation for further studies of their infection phenotypes, host range, and protein functions.

## 2. Materials and Methods

### 2.1. Genome Annotation and Comparative Analysis

The partially sequenced HYPV genome was downloaded from GenBank (accession number: MK860688.1) and subjected to genome annotation. ORFs longer than 50 amino acids (aa) and overlapping with less than 25% with existing ORFs were considered putative genes unless supported otherwise by experimental evidence. Homology searches were initially performed using the EPTV genome (accession number: KY747497) as a reference. If no homologs were identified in EPTV, additional searches were conducted against poxviruses and vertebrates using BLASTp, PSI-BLAST and tBLASTn (https://blast.ncbi.nlm.nih.gov/Blast.cgi accessed between 9 February 2025 and 2 April 2026). Orthopoxvirus gene (OPG) numbers [17] were assigned based on homology to orthopoxvirus genes.

The genome sequences of the two EPTV isolates, the Washington and Saskatoon strains, were compared using MegaBLAST alignment (https://blast.ncbi.nlm.nih.gov/Blast.cgi accessed on 27 February 2026). Regions containing suspected repeat sequences were further analyzed using Tandem Repeat Finder (TRF v4.09) [29] with default parameters.

### 2.2. dN/dS Analysis

Homologous amino acid sequences from EPTV and HYPV were aligned using Clustal Omega (version 1.2.4) [30]. Positive selection analysis was performed using JCoDA v1.4 [31], which generated codon-delimited alignments and calculated dN/dS values through sliding window analysis.

### 2.3. Sequence Alignment and Phylogeny

The homologs of EPTV and HYPV proteins in other poxviruses were identified through BLASTp searches (https://blast.ncbi.nlm.nih.gov/Blast.cgi accessed between 9 February 2025 and 2 April 2026) against representative poxvirus genomes (Table 1). In some cases, in which the homologous genes were not annotated as such, tBLASTn searches and gene synteny analyses were used to identify potential homologs.

The dsRBDs of E3L homologs were identified using InterProScan v109.0 [32] and manually curated. Amino acid sequence alignments were generated using Clustal Omega [30] and MUSCLE v3.8 [33] and visualized in JalView v2.11.5.1 [34].

Maximum-likelihood phylogenetic trees were constructed using PhyML v3.0 with automatic substitution model selection [35,36]. Branch support was assessed using the non-parametric bootstrap analysis with 100 bootstrap replicates. The resulting phylogenetic trees were visualized using FigTree v1.4.3 (https://tree.bio.ed.ac.uk/software/figtree/, accessed on 30 April 2025).

### 2.4. Structural Homology Search and Protein Structure Prediction

EPTV-010.5/181.5 and HYPV-013 were searched for structural homology using the sequence motif search tool in the RCSB Protein Data Bank (https://www.rcsb.org/ accessed on 13 October 2025). Protein structure predictions for VACV, EPTV, and HYPV proteins were generated using the AlphaFold online server powered by AlphaFold 3 [37].

## 3. Results and Discussion

### 3.1. Identification of Previously Unannotated Genes in HYPV

During an initial analysis of HYPV host range proteins, we noticed that several proteins appeared to be absent. Closer inspection revealed that the corresponding ORFs were not annotated in the original study, likely due to a conservative approach that included only ORFs with high sequence identity to EPTV homologs [9]. This resulted in the omission of many ORFs found across multiple other poxvirus species. Therefore, we systematically reanalyzed both genomes to identify additional ORFs. ORFs longer than 50 aa were considered candidate protein-coding regions. ORFs were classified based on a combination of sequence identities with other poxvirus proteins, conserved motifs or domains, gene synteny, and ORF length. ORFs shorter than 50 aa were considered when prior evidence from homologs in other poxviruses indicated the expression and function of the encoded proteins.

**Figure 1:**
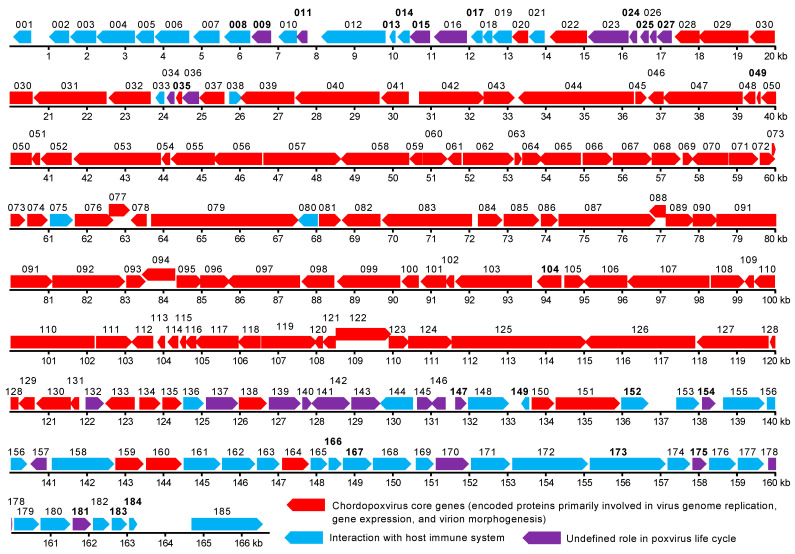
HYPV genome organization. HYPV ORFs are shown as arrows indicating their transcription direction. Core genes involved in the basic replication of poxviruses are shown in red. ORFs whose encoded proteins are implicated in the interaction with the host immune system are shown in blue. Genes with undefined roles in the poxvirus life cycle are shown in purple. ORFs that were newly identified in this study are labeled with bold letters.

We identified 24 previously unannotated ORFs in HYPV (indicated in bold letters in Figure 1), 21 of which have homologs in EPTV or in other poxviruses. The putatively encoded proteins range from 30 to 672 aa, with an average length of 148 aa. ORFs in the HYPV genome were renamed according to their relative position in the genome (HYPV-001 through HYPV-185) (Figure 1, Table 2). The originally identified HYPV ORF names, as well as orthologous ORF names in EPTV, are also shown for reference (Table 2). Three ORFs, HYPV-011 (95 aa), HYPV-147 (95 aa), and HYPV-175 (123 aa), appear to be unique to HYPV since no homologs could be detected in BLASTp or tBLASTn searches. Nine ORFs are specific to the genus *Vespertilionpoxvirus*: HYPV-009, HYPV-015, HYPV-024, HYPV-027, HYPV-034, HYPV-140, HYPV-146, HYPV-154, and HYPV-157.

Three HYPV ORFs lack homologs in EPTV but are found in other poxviruses. HYPV-17 shares 33.67% aa sequence identity with sheeppox virus (SPPV) protein 012, an interleukin-18 binding protein [38]. HYPV-166 shares 26.09% aa sequence identity with MYXV m135, a transmembrane virulence factor [39]. The N-terminal 55 aa of HYPV-181 share 44% aa sequence identity with swinepox virus (SWPV) 009. However, it is restricted to the encoded LAP/PHD finger-like domain; beyond this region, HYPV-181 shows higher sequence identity to other E3 ubiquitin-protein ligases, e.g., *Silurus meridionalis* MARCH1 (accession number: KAI5087763.1).

The original HYPV genome annotation relied on an automated pipeline that only compared homology to EPTV and chose a conservative approach to avoid over-annotating ORFs, by excluding ORFs smaller than 50 codons [9]. However, 22 of the 24 putative ORFs identified here exceed this threshold, and 21 have homologs in other poxviruses.

EPTV contains three unique ORFs not found in other poxviruses, including HYPV: EPTV-143 (74 aa), EPTV-145 (74 aa), and EPTV-176 (62 aa). EPTV-145 consists of 50-nt long tandem repeats containing the consensus pattern 5′-ATG GAC ATG TTT TTT AAA AAG TTT ATT AAT TGT TTT TTT AAA AAA ATT AC-3′, which extend into the adjacent intergenic regions and have a total of five repeat units. In the EPTV isolate Saskatoon/01/2020, this region contains 11 repeat units. Because of the repetitive origin and minor differences in the repeat motif, the predicted aa identity of EPTV-145 between both EPTV isolates is only 53%. Overall, the Washington and Saskatchewan EPTV isolates share 99.7% nucleotide sequence identity and a conserved gene content. Sequence variations are distributed across the genome and no large-scale genomic rearrangements were detected.

In order to analyze if positive selection could be detected in HYPV and EPTV genes, we performed a sliding window analysis with all ORFs pairs in HYPV and EPTV to determine the ratios of non-synonymous to synonymous nucleotide substitutions (dN/dS) (Table 2). dN/dS ratios above 1 are suggestive of positive selection. All the determined dN/dS ratios were found to be below 1, with the highest ratios having values ranging between 0.1 and 0.4. The majority of the ORFs displayed very low dN/dS ratios ranging between 0.003 and 0.09, indicating that a majority of HYPV and EPTV genes have been under strong purifying selection.

### 3.2. Identification of Three Previously Unannotated ORFs in EPTV

We have identified three previously unannotated ORFs in EPTV, which we termed EPTV-010.5/181.5, 031.5, and 045.5 based on their genomic positions between previously annotated genes [26]. The predicted encoded proteins are shorter than 75 aa and have conserved homologs in multiple poxviruses, including HYPV.

#### 3.2.1. Vespertilionpoxviruses Encode Homologs of the STAT1 Antagonist VACV 018

**Figure 2:**
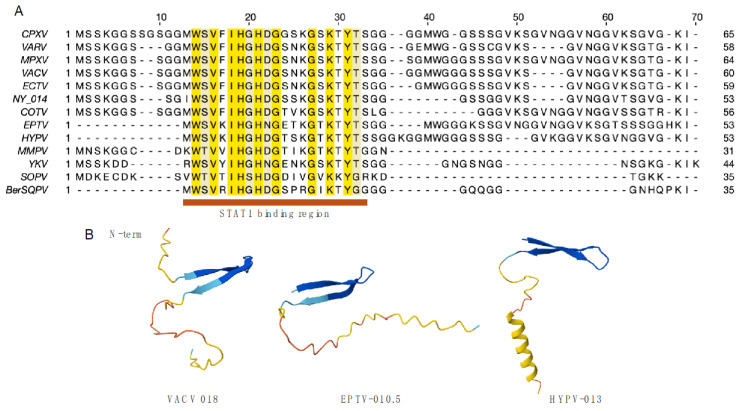
EPTV and HYPV orthologs of VACV WR018, a STAT1 inhibitor. (**A**) Sequence alignment of VACV WR018 orthologs in different poxviruses. Amino acids that are more than 50% conserved are highlighted in yellow, where the conservation is measured based on the number of conserved physicochemical properties for each column of the alignment. The stronger shadings indicate that residues are more conserved at the positions. (**B**) AlphaFold3 structural prediction of VACV, EPTV, and HYPV orthologs. N-termini are on the left. Colors represent the predicted Local Distance Difference Test (pLDDT) of the local regions. Dark blue—pLDDT > 90; light blue—90 > pLDDT > 70; yellow—70 > pLDDT > 50; red—pLDDT < 50.

EPTV-010.5/181.5 and its ortholog HYPV-013 are located near the genomic termini, with the EPTV copies residing within the ITR regions. BLAST searches (v2.17) matched these ORFs to the VACV Western Reserve strain 018 (OPG 024). VACV 018 was shown to antagonize IFN-I- and -II-induced signaling by binding directly to the SH2 domain of signal transducer and activator of transcription 1 (STAT1). The interaction blocks STAT1 phosphorylation, thereby inhibiting its activation and downstream induction of interferon-stimulated genes (ISGs). Although a recombinant VACV Δ018 strain replicated at comparable levels to the WT virus in cell culture, it was attenuated in mice [40]. Many poxviruses encode VACV 018 homologs, which contain a STAT1 binding motif (corresponding to amino acids 11–31 in VACV 018) (Figure 2A). A crystal structure of the STAT1 core and the binding motif of VACV 018 was previously reported and showed that this motif forms a β-hairpin [40]. Prediction of the N-terminal structures of EPTV-010.5 and HYPV-013 using AlphaFold3 is consistent with the reported structure (Figure 2B).

#### 3.2.2. F14.5L Homologs Are Conserved Across Multiple Chordopoxvirus Genera

EPTV-031.5 and its HYPV ortholog HYPV-035 encode proteins of 53 aa in length. To identify the orthologous genes in other species, we first performed BLAST searches with the two proteins, which showed no hits. However, examination of corresponding genomic loci in other poxviruses revealed ORFs of similar length. In the VACV Copenhagen strain, this gene is annotated as F14.5L (OPG 059), located between F14L and F15L. F14.5L has orthologs in other orthopoxviruses [41]. Previous studies showed that the protein product of VACV F14.5L was incorporated into the mature virion (MV) membrane with the C-terminus of the protein exposed on the virion surface, contributing to virion morphology and adhesion of infected cells. Its deletion did not affect VACV replication and MV/EEV production in cell culture but contributed to the virus’s virulence in vivo [41].

**Figure 3:**
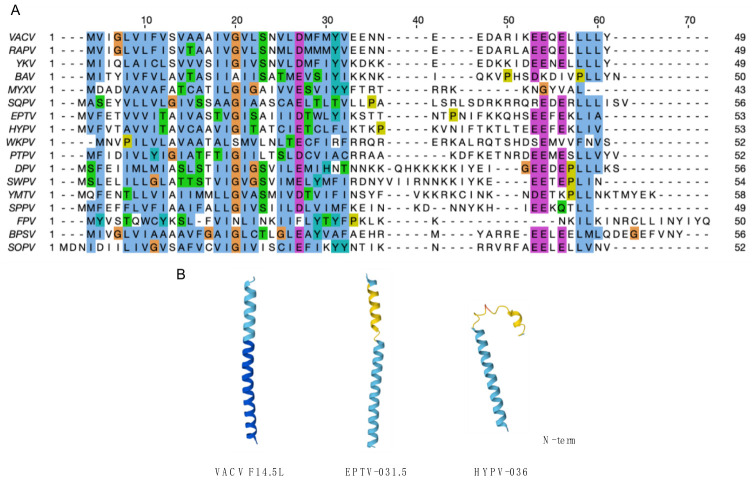
EPTV and HYPV F14.5 orthologs. (**A**) Sequence alignment of F14.5 orthologs in different poxviruses. The amino acids are colored using the Clustal X default coloring based on properties. Blue—hydrophobic; red—positive charge; purple—negative charge; green—polar; pink—cysteine; orange—glycine; yellow—proline; cyan—aromatic; white—non-conserved/gap. (**B**) AlphaFold3 structural prediction of VACV, EPTV, and HYPV F14.5 orthologs. The N-termini are at the bottom. Colors represent the predicted Local Distance Difference Test (pLDDT) of the local regions. Dark blue—pLDDT > 90; light blue—90 > pLDDT > 70; yellow—70 > pLDDT > 50; red—pLDDT < 50.

Homologous ORFs of F14.5L have not been well defined and annotated outside of orthopoxviruses. After identifying homologs in the two vespertilionpoxvirus genomes, we examined the same genomic locus in other chordopoxvirus genera. ORFs of similar length were identified at the syntenic loci in multiple poxviruses (Figure 3A), which share a hydrophobic N-terminus corresponding to a transmembrane region. Structural prediction of VACV, EPTV, and HYPV homologs using AlphaFold3 showed similar α-helical structures at the N-terminus and a more variable C-terminus (Figure 3B). Further studies are needed to determine whether these ORFs encode functional proteins and whether they are expressed and incorporated into MV particles similarly to VACV F14.5L. Although the C-terminus has been implicated in cell morphology and adhesion, its role in virus replication remains unclear.

#### 3.2.3. Entry-Fusion Complex (EFC) Component O3L Homologs

**Figure 4:**
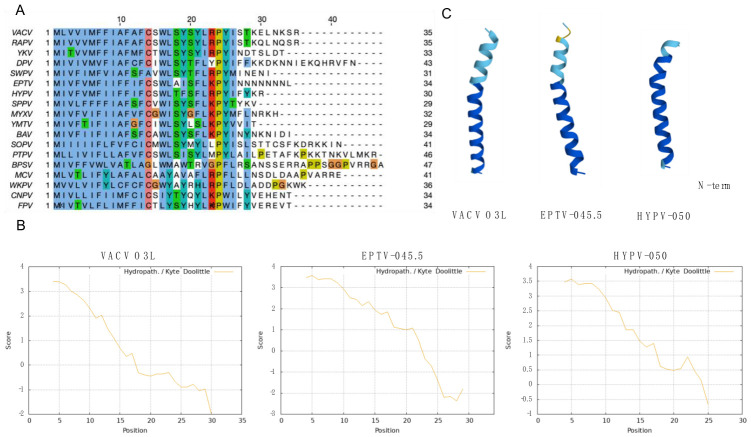
EPTV and HYPV O3 orthologs. (**A**) Sequence alignment of O3 orthologs from different poxviruses. Amino acids were colored using the Clustal X default coloring based on properties. Blue—hydrophobic; red—positive charge; purple—negative charge; green—polar; pink—cysteine; orange—glycine; yellow—proline; cyan—aromatic; white—unconserved/gap. Amino acid residues conserved in all sequences are denoted by asterisks. (**B**) Kyte/Doolittle hydropathy plots of VACV, EPTV, and HYPV O3 orthologs generated by ProtScale. (**C**) AlphaFold3 structural prediction of VACV, EPTV, and HYPV O3 orthologs. The N-termini are at the bottom. Colors represent the predicted Local Distance Difference Test (pLDDT) of the local regions. Dark blue—pLDDT > 90; light blue—90 > pLDDT > 70; yellow—70 > pLDDT > 50; red—pLDDT < 50.

EPTV-045.5 and HYPV-049 are homologs of VACV O3L (OPG 076), whose product, O3, is an integral component of the virus entry-fusion complex (EFC) and the smallest known VACV protein, containing only 35 aa. It is incorporated into the mature virions and is required for virus entry and membrane fusion of infected cells [42]. Deletion of O3L in VACV did not affect progeny virion morphology but significantly reduced infectivity [42]. O3 orthologs exhibit a more conserved N-terminus (aa 1–26) and a more variable C-terminal region (Figure 4A). Of note is a highly conserved proline at position 24, which separates the two α-helices [43,44]. EPTV-045.5 and HYPV-049 are found at genomic locations syntenic to O3L. Hydropathy plots of VACV O3, EPTV-045.5, and HYPV-049 revealed similar profiles, with hydrophobic N-termini and hydrophilic C-termini (Figure 4B). AlphaFold3 structural prediction of VACV O3, EPTV-045.5, and HYPV-049 further indicates similar folding patterns (Figure 4C) and agrees well with the resolved structure of O3 in the EFC [44]. Given that a highly attenuated O3L-deficient VACV could be rescued by O3 homologs from other poxviruses, such as MYXV, SPPV, and fowlpox virus (FWPV) [45], EPTV-045.5 and HYPV-049 are also likely functional components of the EFC.

### 3.3. Gene Fragmentations in Vespertilionpoxviruses

**Figure 5:**
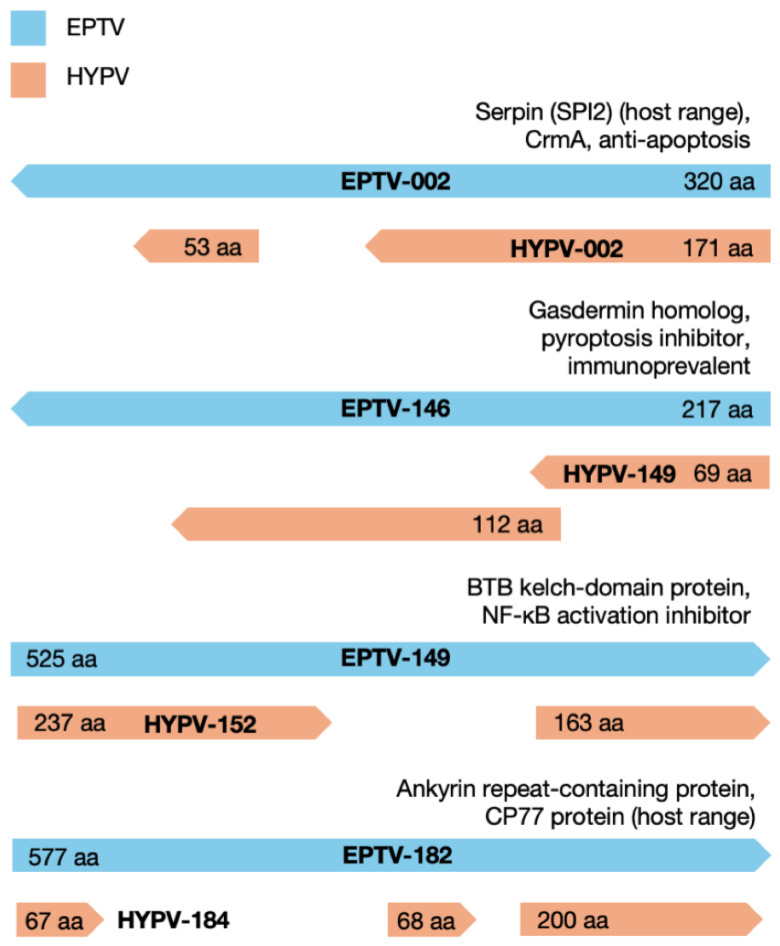
Gene fragmentation in HYPV in comparison to EPTV. EPTV genes are shown in blue and fragmented HYPV genes are shown in orange. The direction of the arrow represent the direction of transcription.

Gene loss through fragmentation is a common theme in poxvirus evolution and may contribute to a more restricted host range [17,18]. Comparison of the HYPV and EPTV genomes revealed four genes that are fragmented in HYPV relative to EPTV: EPTV-002, EPTV-146, EPTV-149, and EPTV-010/182 (corresponding to HYPV-002, HYPV-149, HYPV-152, and HYPV-184, respectively) (Figure 5). These genes encode proteins involved in antagonizing host immune responses and represent potential virulence and host range factors.

EPTV-002 encodes a serine protease inhibitor (serpin). Serpins belong to a large and broadly distributed protein family involved in regulating diverse proteolytic cascades [46]. The number of serpin genes in chordopoxviruses varies from zero in parapoxviruses to four in avipoxviruses [23]. These proteins exhibit anti-apoptotic and anti-inflammatory functions and target host proteins such as cathepsin G, caspases 1, 8 and 10, and granzyme B [47,48,49,50]. Both EPTV and HYPV encode an additional serpin homolog (EPTV-164 and HYPV-168).

EPTV-146 is homologous to the C-terminal regulatory domain of metazoan gasdermins, as demonstrated by the crystal structure of EPTV-146 [51]. Gasdermins are pore-forming effector proteins that act as executioners of pyroptosis, an inflammatory form of induced cell death. Upon activation, inflammasome complexes recruit and cleave gasdermins, releasing the N-terminal lipophilic domain from the C-terminal autoinhibitory domain [52]. The VACV homolog of EPTV-146 was shown to prevent noncanonical pyroptotic cell death and dampen inflammasome signaling by hindering caspase-mediated substrate processing [51]. However, the contribution of these proteins to virus host range and virulence remains unclear. Gene fragmentation or pseudogenization of this locus has also been observed in other poxviruses like CPXV and camelpox virus (CMLV) [51].

EPTV-149 is a BTB kelch-domain protein homologous to VACV A55R. In poxvirus BTB kelch-domain proteins, the N-terminal BTB domain mediates interaction with the Cullin-3-based E3 ubiquitin ligase complex [53], whereas the C-terminal kelch repeats can inhibit NF-κB signaling by targeting host importin α1 [54]. Although fragmented, the corresponding HYPV ORFs retain the full BTB domain and several kelch repeats, making it possible that they are functional. Both EPTV and HYPV encode a second BTB kelch-domain protein (EPTV-155 and HYPV-158).

EPTV-010/182 is an ankyrin repeat-containing protein and homologous to CPXV CP77 and MYXV MT5, two previously identified host range genes [55,56]. CP77 is required for CPXV replication in hamster CHO cells and has been associated with multiple immunomodulatory functions, including inhibition of NF-κB activation and antagonism of the restriction factors SAMD9 and SAMD9L [55,57,58]. In addition to the fragmented HYPV-184, HYPV encodes two additional CP77 homologs, HYPV-012 and HYPV-173. Notably, EPTV-010/182 and HYPV-012 are approximately 100 aa shorter at the C-terminus than their CPXV homolog and thus lack carboxy-terminal F-box-like domains, whereas HYPV-173 is full length.

### 3.4. Gene Duplications in Vespertilionpoxviruses

**Figure 6:**
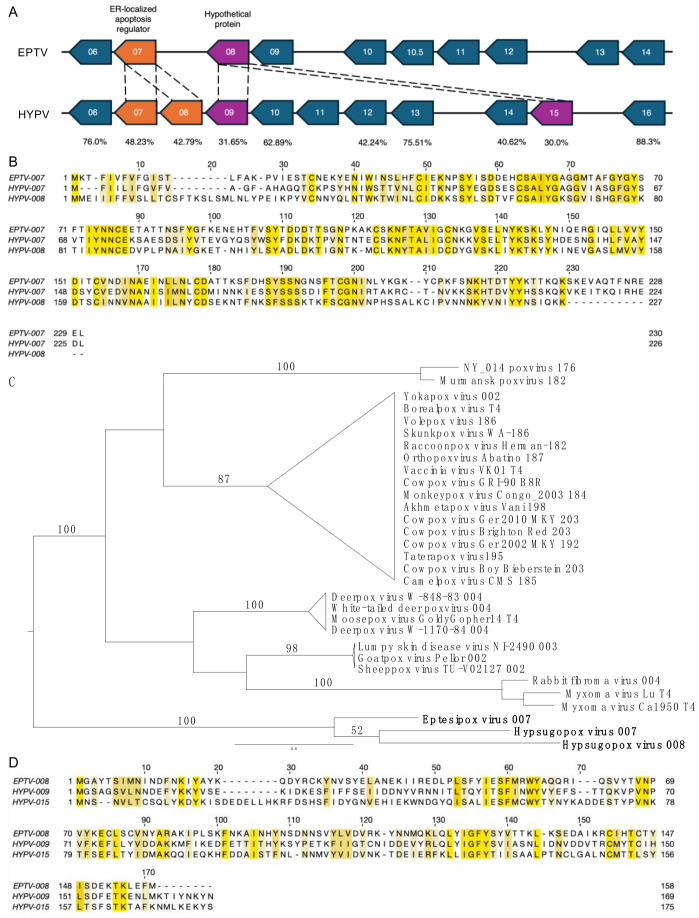
Gene duplications in HYPV. (**A**) A schematic representation of the genomic locations of the duplicated HYPV ORFs. The two sets of duplicated ORFs are shown in orange or purple. At the bottom are the percent aa identities of HYPV ORFs with their EPTV homologs. (**B**) Sequence alignment of EPTV-007 and its two HYPV homologs, HYPV-007 and HYPV-008. Amino acids that are 50% conserved are highlighted in yellow, where the conservation is measured based on the number of conserved physicochemical properties for each column of the alignment. The stronger shadings indicate the residues are more conserved at the positions. (**C**) Phylogenetic tree of MT4 homologs. The tree is midpoint-rooted, and bootstrap support of ≥50 is indicated above the branches. The EPTV and HYPV MT4 homologs are shown in bold. (**D**) Sequence alignment of EPTV-008 and its two HYPV homologs, HYPV-009 and HYPV-016, with the same highlight as in (**B**).

Gene duplications in poxviruses contribute to functional diversification and adaptation. Two EPTV genes in the same genomic region, EPTV-007 and EPTV-008, are duplicated in HYPV (Figure 6A).

#### 3.4.1. Gene Duplications in HYPV

EPTV-007, HYPV-007, and HYPV-008 are homologs of M-T4-like apoptosis inhibitors. The MYXV ortholog, M-T4, is a recognized host range factor because induced apoptosis was observed in rabbit RL5 cells and peripheral blood lymphocytes infected with T4-deleted MYXV, but not in RK13 cells [59]. The cowpox ortholog, CPXV203 (OPG 195), binds fully assembled MHC I proteins and retrieves them to the ER via its C-terminal ER retrieval sequence (KTEL). Therefore, it could downregulate MHC I and reduce detection of infected cells by CD8+ T cells [60,61]. The C-terminal ER retrieval signal is retained in EPTV-007 and HYPV-007 (Figure 6B). This motif mediates retention of proteins in the ER through continuous retrieval of proteins from the Golgi apparatus [62,63]. In contrast, HYPV-008 lacks the C-terminal 13 aa and has lost this signal peptide (Figure 6B). Phylogenetic analysis shows that EPTV and HYPV M-T4 orthologs cluster more closely with those of other clade-II poxviruses, forming a clade distinct from orthopoxvirus and centapoxvirus homologs (Figure 6C).

The other duplicated HYPV ORFs, HYPV-009 and HYPV-015, share 29.7% aa sequence identity and show no detectable matches in BLAST analyses (v2.17) other than their EPTV ortholog, but are predicted to contain Bcl-2-like domains (Figure 6D). Bcl-2-like domains are present in several poxvirus proteins, including VACV B14, A46, A52, C1, K7, N1, and N2. These proteins are involved in inhibiting the Toll-like receptor signaling pathway and exhibit anti-apoptotic and anti-inflammatory activities [64,65]. However, due to low sequence similarity, the precise relationship between vespertilionpoxvirus proteins and other poxvirus Bcl-2-like proteins remains unclear.

#### 3.4.2. Presence of Two E3L Homologs in EPTV

A unique genomic feature of EPTV among currently characterized poxviruses is the presence of two homologs of the dsRNA-binding protein kinase R (PKR) inhibitor E3L (OPG 65) (EPTV-037 and EPTV-163) in distinct regions of the genome [26]. VACV E3L is a well-established virulence and host range gene. It consists of an N-terminal Zα domain and a C-terminal dsRNA-binding domain (dsRBD), which bind Z-form nucleic acids and dsRNA, respectively. E3 inhibits multiple innate immunity pathways triggered by viral nucleic acids, including the PKR pathway, the 2′-5′-oligoadenylate synthetase (OAS)/RNase L pathway, RIG-I/MDA5-mediated IFN induction, and Z-DNA binding protein 1 (ZBP1)-mediated IFN signaling and necroptosis [66,67,68]. While the Zα domain of VACV E3L is dispensable for virus replication in cell culture, both domains are necessary for virus full pathogenesis in mouse models [69,70,71]. E3L homologs are found in many poxviruses. However, some of them lack a functional Zα domain, as observed in MYXV, rabbit fibroma virus (RFV), and MPXV [23]. In addition to its role in virulence, E3L has been described as a host range gene, because it is dispensable for VACV infection in Syrian hamster cells but required in human cells [72]. A molecular explanation for this difference is that Syrian hamster PKR is resistant to E3 inhibition but sensitive to the alternative VACV PKR inhibitor K3, whereas human PKR is sensitive to E3 but resistant to K3 [73]. The only other poxvirus known to have two E3 orthologs is cetacean poxvirus 1 (CePV-1), which contains two closely related, tandemly arranged homologs (CePV-TA-20 and CePV-TA-21). Whereas CePV-TA-21 encodes both Zα and dsRBDs, CePV-TA-20 contains a dsRBD but lacks the Zα domain [74]. In contrast, the two EPTV E3 homologs are located in distinct regions of the genome. Although both homologs contain Zα and dsRBD domains, they share only 22% aa sequence identity. Uniquely among E3 homologs, EPTV-163 contains an 80 aa C-terminal extension with no detectable homology to known proteins.

**Figure 7:**
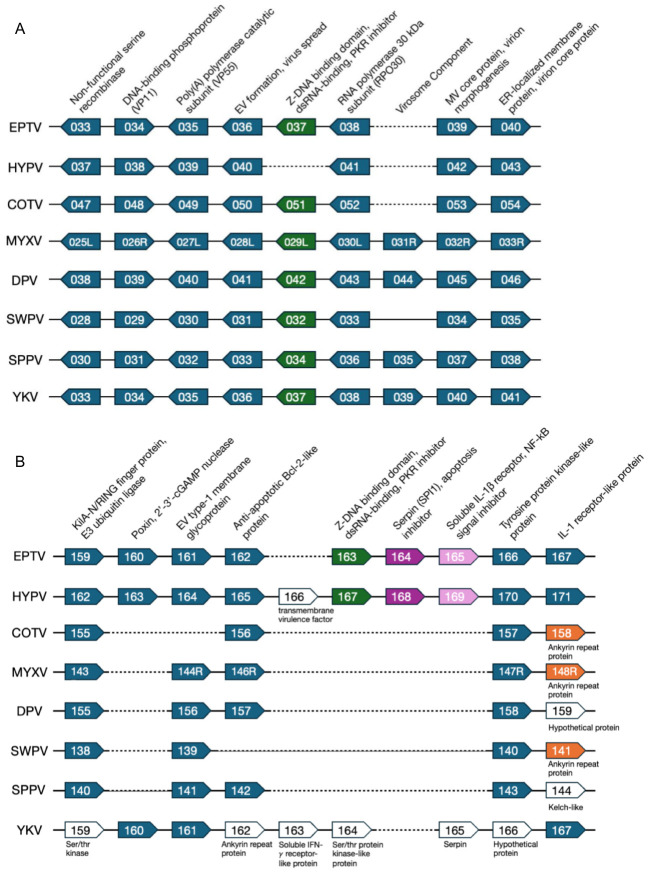
Synteny conservation around the E3L homologs EPTV-037 (**A**) and EPTV-163 (**B**). The arrows on the horizontal line represent genes, with the direction corresponding to the transcription orientation. Homologous genes in different genomes are aligned vertically with the same color. E3L orthologs are highlighted in green. Genes without orthologs in the examined genomes are highlighted in white.

**Figure 8:**
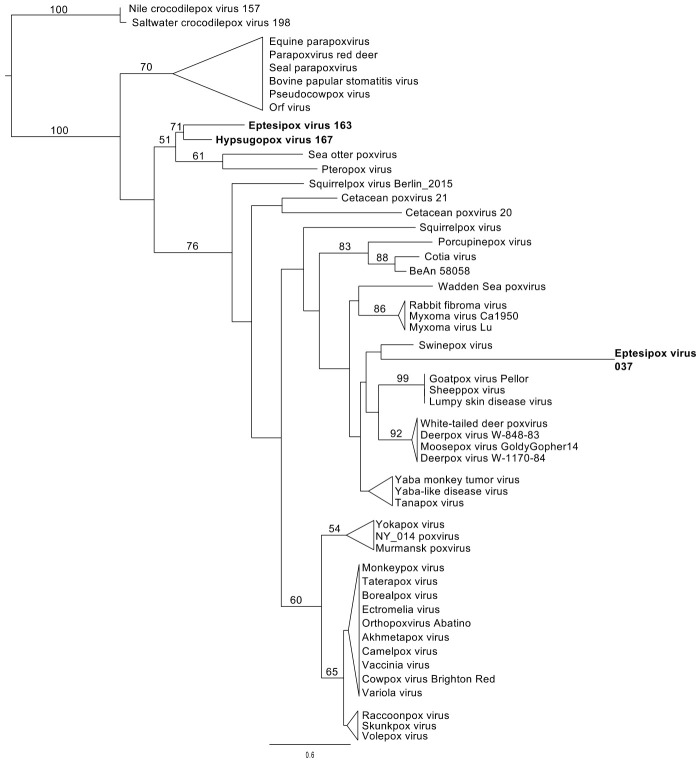
Phylogenetic relationship between poxviral E3 homologs. The dsRBD regions of E3 homologs and crocodilepox virus 157, a distantly related dsRNA-binding protein [75], were aligned using MUSCLE and used for constructing the phylogenetic tree using PhyML. The tree was rooted to crocodilepox virus 157 and saltwater crocodilepox virus 198. Bootstrap support of ≥50 is indicated above the branches. The EPTV and HYPV E3 homologs are indicated in bold. The branches of clades were collapsed and shown as triangles for better clarity.

Synteny analyses of the loci containing the two EPTV E3 homologs revealed that EPTV-037 occupies the same genomic location as E3 homologs in other clade II poxviruses (Figure 7A). We identified a single, previously unannotated E3L homolog in HYPV (HYPV-169), which is syntenic to EPTV-163 (Figure 7B). Phylogenetic analysis based on the conserved dsRBD of E3Ls shows that EPTV-037 and EPTV-163 cluster in distinct clades, confirming that they are not closely related (Figure 8). Consistent with the synteny analysis, EPTV-163 and HYPV-169 are closely related, indicating that they are orthologs. These observations indicate that the common ancestor of EPTV and HYPV acquired a second E3 homolog from an unidentified poxvirus rather than through duplication of the ancestral EPTV-037. HYPV subsequently lost the ancestral copy, leaving only HYPV-169. Future studies are required to determine whether the two EPTV E3L homologs have diverged functionally and whether retention of both copies confers adaptive advantages.

### 3.5. Other Host Range Genes in Vespertilionpoxviruses

As previously noted, EPTV contains 11 of 12 described host range factors, lacking only K1L, which is specific to orthopoxviruses and centapoxviruses [26]. HYPV also encodes orthologs of these proteins. However, in addition to those described above, several vespertilionpoxvirus host range genes exhibit distinct features compared with their counterparts in other poxviruses.

VACV K3 (encoded by K3L, OPG 041) is another inhibitor of the PKR pathway, and its ortholog is newly annotated in HYPV (HYPV-014 and EPTV-012). K3 acts as a structural mimic of eIF2α and antagonizes the PKR pathway by binding directly to activated PKR, thereby preventing its interaction with its substrate [76,77]. A K3L-deleted VACV strain showed reduced replication in mouse L929 cells and hamster BHK cells but was unaffected in human HeLa and rabbit RK13 cells [72,76,78]. Notably, the EPTV K3L homolog encodes an unusual C-terminal extension that is not observed in other poxviruses [79]. In contrast, the HYPV K3L homolog lacks this extension, indicating that it is unique to EPTV. Previous studies in yeast have shown that the C-terminal extension modulated the inhibitory activity of EPTV K3L in a species-specific manner [79]; however, its role in mediating interactions with PKR remains unclear.

EPTV-177 and HYPV-183 (199 aa and 131 aa in length, respectively) are homologs of cellular tumor necrosis factor receptors (TNFRs). Poxviral TNFR homologs can bind TNF cytokines, inhibiting the downstream signaling pathway involved in regulating host inflammatory responses and apoptosis [80,81]. Orthopoxviruses can encode up to four TNFRs, designated as CrmB, CrmC, CrmD, and CrmE in CPXV. Among clade II poxviruses, only leporipoxviruses encode TNFR homologs, called MT2 in MYXV. This protein contains four N-terminal cysteine-rich domains (CRDs) homologous to mammalian TNFR CRDs, as well as a poxvirus-specific C-terminal domain involved in the secretion of the protein [82]. EPTV-177 is truncated relative to the MYXV T2 homolog (326 aa), lacking the C-terminal domain, whereas HYPV-183 is further truncated and contains only two N-terminal CRDs. Similar truncations have been observed in some orthopoxvirus strains. Notably, a truncated MYXV T2 containing only two CRDs was unable to bind TNF but still inhibited apoptosis [82].

Another group of host range factors belongs to a family of poxviral proteins containing short consensus repeats (SCRs), also known as complement control protein modules, which are often involved in the regulation of the complement system. Most orthopoxviruses and centapoxviruses contain two SCR-encoding genes, called B5R (OPG 190) and C3L (OPG 32) in VACV [23]. B5R has four tandemly arranged SCRs and a C-terminal transmembrane domain (TM). It is expressed on the surface of the enveloped virion as a membrane glycoprotein and is essential for extracellular enveloped virus formation and actin tail-mediated virion repulsion [83,84]. C3L also contains four SCRs but lacks the TM domain. It is secreted and inhibits the complement system by interacting with C3b and C4b [85]. Clade II poxviruses typically encode a single B5R-like gene. In yatapoxviruses, leporipoxviruses, and deerpoxviruses, these proteins consist of three SCRs and a TM domain, whereas in capripoxviruses and suipoxviruses, they contain two SCRs and a TM domain [23]. Interestingly, both EPTV and HYPV encode two B5R-like proteins. EPTV-161 and HYPV-164 (237 aa and 235 aa, respectively) resemble the domain organization of B5R-like orthologs from capripoxviruses and suipoxviruses. On the other hand, the domain organization of EPTV-174 and HYPV-180 (263 aa and 260 aa, respectively) is more similar to that of VACV C3L, containing four SCRs and no TM domain. However, whether these proteins function similarly to C3L remains unknown. Phylogenetic and sequence analyses of the B5R-like protein family suggest that the two copies in orthopoxvirus originated through a duplication event early in the genus [23]. The presence of apparent C3L-like orthologs in vespertilionpoxviruses raises additional questions about their evolutionary history and warrants further investigation.

## 4. Conclusions

In this study, we analyzed the genomes of two bat-infecting poxviruses from the genus *Vespertilionpoxvirus*, identified previously unannotated ORFs, and conducted a comparative analysis of their gene content. We identified 24 previously unannotated ORFs in HYPV and three in EPTV, substantially expanding the number of ORFs encoded by their genomes. Comparative genomic analyses further revealed multiple gene duplication, deletion, and fragmentation events, especially in genes implicated in virulence and host range functions. These findings demonstrate that the genomes of vespertilionpoxviruses are more complex and dynamic than previously recognized and provide new insights into their genomic organization and evolutionary history. Some of these genomic changes may contribute to adaptation to their respective bat host.

Our results also highlighted the importance of thorough manual curation of poxvirus genomes to ensure accurate annotation. Although automated pipelines for poxvirus genome annotation are readily available [86], many poxvirus genes are highly divergent and subject to active adaptation during host–virus interactions. As a result, the assignment of homologous relationships should not solely rely on sequence identity but should integrate additional evidence, including genomic context (synteny), conserved domains, and structural similarity, taking advantage of the fast-developing protein structure prediction tools. This integrative approach is particularly important for identifying short ORFs and highly divergent genes that are often missed or misannotated by automated methods.

We also identified sequence variations in several host range genes in both HYPV and EPTV. These genes have been previously implicated in determining the range of host cells that poxviruses can infect and play a role in host tropism and viral adaptation [22,23,24]. Although no clear signs of positive selection were observed between HYPV and EPTV, the variation may reflect adaptation to specific bat hosts. Functional characterization of these genes will be important to determine how these genomic differences influence viral replication, immune evasion, and host specificity.

A major limitation in the study of bat poxviruses is the lack of well-established experimental systems. At present, most knowledge is derived from field observations, pathology reports, and full or partial genome sequences. The development of robust in vitro and in vivo models will be critical for validating the expression and functions of identified putative ORFs, understanding the biological significance of the genomic differences, and advancing our understanding of *Vespertilionpoxvirus* biology and zoonotic potential.

## Figures and Tables

**Table 1:** Viruses used in the analysis and their genome accession number.

Abbreviation	Virus Name	Accession Number
BAV	BeAn 58058 virus	KY094066.1
BPSV	Bovine papular stomatitis virus	AY386265.1
CNPV	Canarypox virus	AY318871.1
COTV	Cotia virus	HQ647181.2
CPXV	Cowpox virus	LT883663.1
DPV	Deerpox virus	AY689436.1
ECTV	Ectromelia virus	NC_004105.1
EPTV	Eptesipox virus	KY747497.1
FPV	Fowlpox virus	AF198100.1
HYPV	Hypsugopox virus	MK860688.1
MCV	Molluscum contagiosum virus	U60315.1
MPXV	Monkeypox virus	MT903340.1
MMPV	Murmansk poxvirus	MF001304.1
MYXV	Myxoma virus	AF170726.2
NY_014	NY_014 poxvirus	MF001305.1
PTPV	Pteropox virus	KU980965.1
RAPV	Raccoonpox virus	KP143769.1
SOPV	Sea otterpox virus	MH427217.1
SPPV	Sheeppox virus	AY077832.1
SQPV	Squirrelpox virus	HE601899.1
BerSQPV	Squirrelpox virus Berlin	MF503315.1
SWPV	Swinepox virus	AF410153.1
VACV	Vaccinia virus	AY243312.1
VARV	Variola virus	DQ441416.1
WKPV	Western grey kangaroopox virus	MF467280.1
YMTV	Yaba monkey tumor virus	AY386371.1
YKV	Yokapox virus	HQ849551.1

**Table 2:** Updated HYPV genome annotation.

HYPV								
New HYPV ORF Name	Old HYPV ORF Name	ORF Position	aa Length	Homologous EPTV ORF ^1^	% aa Identity	Putative Protein Function	dN/dS Ratio	OPG ^2^
HYPV-001	HYPV-1	557-87	157	**EPTV-001**	35.71	Soluble IL-1β receptor, NF-kB signal inhibitor	0.1202	200
HYPV-002	HYPV-2	1552-1037	172	**EPTV-002**		Serpin (SPI2) (host range), CrmA, anti-apoptosis (partial)		199
HYPV-003	HYPV-3	2261-1581	227	**EPTV-003**	69.47	Bcl-2 domain, α-amanitin target protein, nuclear IRF3 inhibitor	0.1118	36
HYPV-004	HYPV-4	3316-2309	336	**EPTV-004**	46.81	IL-1 receptor-like protein	0.1195	
HYPV-005	HYPV-5	3835-3356	160	**EPTV-005**	75.95	TLR-induced NF-κB pathway inhibitor	0.0473	
HYPV-006	HYPV-6	4774-3872	301	**EPTV-006**	76	Tyrosine protein kinase-like protein	0.0406	
HYPV-007	HYPV-7	5522-4842	227	**EPTV-007**	48.23	ER-localized apoptosis regulator (host range), retains MHC I in ER	0.1436	195
HYPV-008	-	6303-5620	227	**EPTV-007**	42.79	ER-localized apoptosis regulator (host range), retains MHC I in ER	0.1325	195
HYPV-009	-	6882-6373	169	**EPTV-008**	31.65	Hypothetical protein	0.3381	
HYPV-010	HYPV-8	7481-7002	159	**EPTV-009**	62.89	Soluble IL-1β receptor, NF-kB signal inhibitor	0.1522	200
HYPV-011	-	7771-7484	95			Hypothetical protein		
HYPV-012	HYPV-9	9826-8141	561	**EPTV-010**	42.24	Ankyrin repeat-containing protein	0.1112	23
HYPV-013	-	10,078-9917	53	EPTV-010.5 *	75.51	STAT1 binding, type I IFN inhibitor	0.1091	24
				**EPTV-011**		Ankyrin repeat-containing protein		
HYPV-014	-	10,421-10,131	96	**EPTV-012**	40.62	eIF2a-like PKR inhibitor (host range)	0.1594	41
HYPV-015	-	10,992-10,465	175	**EPTV-008**	30	Hypothetical protein	0.2229	
				**EPTV-013**		Ankyrin repeat-containing protein (partial)		
HYPV-016	HYPV-10	11,913-11,053	286	EPTV-014	88.3	Monoglyceride lipase homolog	0.0205	43
HYPV-017	-	12,336-12,037	99	SPPV-012	33.67	IL-18 binding protein		
HYPV-018	HYPV-11	12,588-12,340	82	EPTV-015	54.43	Secreted EGF-like growth factor	0.0035	19
HYPV-019	HYPV-12	13,100-12,594	168	EPTV-016	47.27	Anti-apoptotic factor, mitochondrial-associated (host range)	0.1143	45
HYPV-020	HYPV-13	13,569-13,144	141	EPTV-017	73.76	dUTPase	0.0531	46
HYPV-021	HYPV-14	14,004-13,597	135	EPTV-018	69.4	Pyrin-domain containing protein, inhibits inflammasome activation (host range)	0.0716	
HYPV-022	HYPV-15	15,034-14,060	324	EPTV-019	88.54	Ribonucleotide reductase small subunit	0.0183	48
HYPV-023	HYPV-16	16,139-15,075	354	EPTV-020	56.98	Immunoglobulin domain, type I membrane protein	0.0776	49
HYPV-024	-	16,361-16,161	66	EPTV-021	34.85	Hypothetical protein	0.4167	
HYPV-025	-	16,646-16,425	73	EPTV-022	43.66	Hypothetical protein	0.1766	51
HYPV-026	HYPV-17	16,869-16,687	60	EPTV-023	58.33	Cytoplasmic protein, protein with iActA-like proline repeats	0.0671	52
HYPV-027	-	17,327-16,941	128	EPTV-024	49.58	Hypothetical protein	0.1281	
HYPV-028	HYPV-18	18,008-17,361	215	EPTV-025	80	Entry-fusion complex (EFC) component, S-S bond formation pathway protein substrate	0.0423	53
HYPV-029	HYPV-19	19,314-17,998	438	EPTV-026	89.47	Ser/Thr protein kinase	0.0227	54
HYPV-030	HYPV-20	20,626-19,334	430	EPTV-027	76.05	RhoA-mDia signaling inhibitor	0.0365	55
HYPV-031	HYPV-21	22,602-20,659	647	EPTV-028	76.78	EV maturation protein	0.0323	56
HYPV-032	HYPV-22	23,755-22,640	371	EPTV-029	95.69	Palmitoylated enveloped virion membrane glycoprotein, phospholipase D-like	0.0067	57
HYPV-033	HYPV-23	24,008-23,781	75	EPTV-030	45.33	NF-κB activation inhibitor	0.234	58
HYPV-034	HYPV-24	24,250-24,050	66	EPTV-031	92.42	Hypothetical protein	0.0249	
HYPV-035	-	24,414-24,253	53	EPTV-031.5 *	47.17	MV membrane protein, cell adhesion	0.2472	59
HYPV-036	HYPV-25	24,917-24,471	148	EPTV-032	80.41	Hypothetical protein	0.0567	60
HYPV-037	HYPV-26	25,654-24,992	220	EPTV-033	67.27	Non-functional serine recombinase	0.1251	61
HYPV-038	HYPV-27	25,714-26,052	112	EPTV-034	82.69	DNA-binding phosphoprotein (VP11)	0.0404	62
HYPV-039	HYPV-28	27,461-26,046	471	EPTV-035	84.71	Poly (A) polymerase catalytic subunit (VP55)	0.0265	63
HYPV-040	HYPV-29	29,676-27,478	732	EPTV-036	83.47	EV formation, virus spread	0.0429	64
				EPTV-037		Z-DNA binding domain, dsRNA-binding, PKR inhibitor (host range)		65
HYPV-041	HYPV-30	30,455-29,733	240	EPTV-038	86.25	RNA polymerase 30 kDa subunit (RPO30)	0.0222	66
HYPV-042	HYPV-31	30,760-32,463	567	EPTV-039	88.54	MV core protein, virion morphogenesis	0.0268	68
HYPV-043	HYPV-32	32,490-33,302	270	EPTV-040	91.48	ER-localized membrane protein, virion core protein	0.0257	70
HYPV-044	HYPV-33	36,319-33,299	1006	EPTV-041	86.88	DNA polymerase, catalytic subunit	0.0156	71
HYPV-045	HYPV-34	36,352-36,642	96	EPTV-042	87.5	Sulfhydryl oxidase (FAD-linked), S-S bond formation pathway, virion protein	0.0248	72
HYPV-046	HYPV-35	37,055-36,645	136	EPTV-043	80.88	MV core protein	0.0326	73
HYPV-047	HYPV-36	39,117-37,039	692	EPTV-044	81.91	Virulence, membrane protein, activates of ERK1/2 signaling pathway	0.0237	74
HYPV-048	HYPV-37	39,487-39,173	104	EPTV-045	82.69	Glutaredoxin, ribonucleotide reductase cofactor	0.0404	75
HYPV-049	-	39,597-39,505	30	EPTV-045.5 *	76.67	MV membrane, Entry/fusion complex component	0.0579	76
HYPV-050	HYPV-38	40,545-39,613	310	EPTV-046	82.58	DNA-binding core protein, virosomal protein, winged HTH domain	0.0277	77
HYPV-051	HYPV-39	40,767-40,546	73	EPTV-047	76.71	MV membrane protein required for morphogenesis and entry	0.0905	78
HYPV-052	HYPV-40	41,577-40,768	269	EPTV-048	77.82	ssDNA-binding phosphoprotein	0.04	79
HYPV-053	HYPV-41	43,925-41,640	761	EPTV-049	86.58	Ribonucleotide reductase large subunit	0.0171	80
HYPV-054	HYPV-42	44,202-43,966	78	EPTV-050	75.64	MV protein (VP13)	0.0508	81
HYPV-055	HYPV-43	45,371-44,220	383	EPTV-051	76.9	Telomere binding protein, DNA packaging	0.0321	82
HYPV-056	HYPV-44	46,650-45,364	428	EPTV-052	80.84	Virion core cysteine protease, required for morphogenesis	0.0265	83
HYPV-057	HYPV-45	46,656-48,686	676	EPTV-053	85.8	Core DNA/RNA-dependent NTPase (NPH-II), DNA and RNA helicase	0.0184	84
HYPV-058	HYPV-46	50,465-48,678	595	EPTV-054	83.87	Metalloprotease-like protein	0.0213	85
HYPV-059	HYPV-47	50,794-50,462	110	EPTV-055	90	MV membrane, Entry/fusion complex component	0.0909	86
HYPV-060	HYPV-48	50,788-51,456	222	EPTV-056	77.48	Late transcription elongation factor (VLTF)	0.0484	87
HYPV-061	HYPV-49	51,800-51,423	125	EPTV-057	68	Glutaredoxin S-S bond formation pathway; thioredoxin-like	0.1469	88
HYPV-062	HYPV-50	51,803-53,140	445	EPTV-058	76.71	Fen-1-like nuclease, DNA repair and recombination	0.037	89
HYPV-063	HYPV-51	53,142-53,333	63	EPTV-059	88.89	RNA polymerase subunit (RPO7)	0.0473	90
HYPV-064	HYPV-52	53,337-53,870	177	EPTV-060	79.1	NLPc/P60 superfamily protein, predicted hydrolase	0.0277	91
HYPV-065	HYPV-53	54,933-53,836	365	EPTV-061	79.06	Virion phosphoprotein, early morphogenesis	0.03	92
HYPV-066	HYPV-54	54,962-55,744	260	EPTV-062	96.54	Viral late transcription factor 1 (VLTF-1), PCNA homolog	0.0062	93
HYPV-067	HYPV-55	55,760-56,782	340	EPTV-063	83.24	MV membrane, myristylated entry/fusion complex component	0.0305	94
HYPV-068	HYPV-56	56,783-57,532	249	EPTV-064	89.16	MV membrane, myristylated entry/fusion complex component	0.0152	95
HYPV-069	HYPV-57	57,558-57,833	91	EPTV-065	68.54	crescent membrane, viral membrane assembly proteins	0.081	96
HYPV-070	HYPV-58	58,790-57,825	321	EPTV-066	84.69	Internal virion protein, required for early transcription by cores	0.0283	97
HYPV-071	HYPV-59	58,815-59,573	252	EPTV-067	93.25	DNA-binding core transcription protein (VP8), early mRNA regulation	0.0186	98
HYPV-072	HYPV-60	59,588-59,992	134	EPTV-068	82.84	MV membrane, entry/fusion complex component	0.1358	99
HYPV-073	HYPV-61	59,934-60,380	148	EPTV-069	86.49	Virion membrane protein, early stage morphogenesis	0.0918	100
HYPV-074	HYPV-62	60,402-60,932	176	EPTV-070	80.68	Thymidine kinase	0.0306	101
HYPV-075	HYPV-63	61,026-61,625	199	EPTV-071	60.41	Type I IFN inhibitor (host range), antagonist of SAMD9, and SAMD9L	0.0026	27
HYPV-076	HYPV-64	61,692-62,693	333	EPTV-072	87.39	Poly (A) polymerase small subunit (VP39), cap methyltransferase	0.0554	102
HYPV-077	HYPV-65	62,608-63,165	185	EPTV-073	86.49	RNA polymerase subunit (RPO22)	0.0306	103
HYPV-078	HYPV-66	63,580-63,170	136	EPTV-074	81.48	MV membrane, entry/fusion complex component	0.0332	104
HYPV-079	HYPV-67	63,688-67,545	1285	EPTV-075	94.32	RNA polymerase subunit (RPO147)	0.0063	105
HYPV-080	HYPV-68	68,060-67,542	172	EPTV-076	90.7	Tyr/Ser phosphatase, IFN-γ inhibitor, dephosphorylates STAT1	0.0174	106
HYPV-081	HYPV-69	68,074-68,646	190	EPTV-077	92.63	MV membrane, entry/fusion complex component	0.023	107
HYPV-082	HYPV-70	69,667-68,654	337	EPTV-078	79.46	MV heparin bind surface protein	0.0197	108
HYPV-083	HYPV-71	72,058-69,671	795	EPTV-079	92.45	RNA polymerase-associated protein (RAP94), early transcription	0.0173	109
HYPV-084	HYPV-72	72,228-72,872	214	EPTV-080	61.95	Late transcription factor 4 (VTLF-4), multifunctional protein	0.0792	110
HYPV-085	HYPV-73	72,894-73,829	311	EPTV-081	83.6	DNA topoisomerase type I	0.0538	111
HYPV-086	HYPV-74	73,868-74,314	148	EPTV-082	77.03	Crescent membrane and IV formation	0.0701	112
HYPV-087	HYPV-75	74,355-76,889	844	EPTV-083	87.91	mRNA capping enzyme large subunit, transcription termination factor	0.0146	113
HYPV-088	HYPV-76	77,288-76,851	145	EPTV-084	77.93	Virion core protein, early stage morphogenesis	0.0974	114
HYPV-089	HYPV-77	77,287-78,030	247	EPTV-085	68.02	Virion core protein, early stage morphogenesis	0.1698	115
HYPV-090	HYPV-78	78,027-78,683	218	EPTV-086	91.28	Uracil DNA glycosylase, DNA pol processivity factor	0.0222	116
HYPV-091	HYPV-79	78,717-81,080	787	EPTV-087	91.11	NTPase, DNA primase, and nucleic acid-independent nucleoside triphosphatase	0.0115	117
HYPV-092	HYPV-80	81,077-82,984	635	EPTV-088	96.69	Early transcription factor small subunit (VETF-s), ATPase, predicted helicase	0.0046	118
HYPV-093	HYPV-81	83,017-83,532	171	EPTV-089	78.82	RNA polymerase subunit (RPO18)	0.0384	119
HYPV-094	HYPV-82	84,339-83,464	291	EPTV-090	68.86	Carbonic anhydrase, GAG-binding MV membrane protein	0.0928	120
HYPV-095	HYPV-83	84,397-85,068	223	EPTV-091	76.02	mRNA decapping enzyme	0.0428	121
HYPV-096	HYPV-84	85,043-85,822	259	EPTV-092	84.5	mRNA decapping enzyme, mitochondrial	0.0291	122
HYPV-097	HYPV-85	87,703-85,796	635	EPTV-093	94.17	DNA-dependent ATPase (NPH-I), transcription termination	0.008	123
HYPV-098	HYPV-86	88,609-87,746	287	EPTV-094	89.55	mRNA capping enzyme small subunit, transcription initiation factor	0.0154	124
HYPV-099	HYPV-87	90,295-88,643	550	EPTV-095	90.91	Trimeric virion coat protein (rifampicin resistance)	0.0114	125
HYPV-100	HYPV-88	90,776-90,321	151	EPTV-096	82.78	Late transcription factor (VLTF-2)	0.0282	126
HYPV-101	HYPV-89	91,479-90,805	224	EPTV-097	95.98	Late transcription factor (VLTF-3)	0.0058	127
HYPV-102	HYPV-90	91,706-91,476	76	EPTV-098	89.47	Virion protein, S-S bond formation pathway	0.067	128
HYPV-103	HYPV-91	93,726-91,726	666	EPTV-099	83.81	Precursor of major core protein 4b (p4b), morphogenesis	0.0228	129
HYPV-104	-	94,424-93,780	214	EPTV-100	42.62	Membrane-associated virion core protein (p39), morphogenesis	0.1317	130
HYPV-105	HYPV-92	94,462-94,992	176	EPTV-101	79.88	RNA polymerase subunit (RPO19)	0.0299	131
HYPV-106	HYPV-93	96,107-94,989	372	EPTV-102	85.75	Crescent membrane and IV formation, VMAP	0.0217	132
HYPV-107	HYPV-94	98,275-96,131	714	EPTV-103	91.88	Early transcription factor large subunit (VETF-L)	0.0135	133
HYPV-108	HYPV-95	98,338-99,213	291	EPTV-104	86.6	Intermediate transcription factor small subunit (VITF-3s)	0.0205	134
HYPV-109	HYPV-96	99,459-99,223	78	EPTV-105	87.18	MV membrane, early morphogenesis	0.0461	135
HYPV-110	HYPV-97	102,192-99,460	910	EPTV-106	76.87	Precursor of major core protein 4a (p4a), morphogenesis	0.0297	136
HYPV-111	HYPV-98	102,207-103,142	311	EPTV-107	91	Crescent membrane and IV formation, VMAP	0.0136	137
HYPV-112	HYPV-99	103,705-103,139	188	EPTV-108	69.82	Virion core protein, morphogenesis	0.0651	138
HYPV-113	HYPV-100	104,002-103,139	287	EPTV-109	62.12	MV membrane phosphoprotein, morphogenesis	0.05	139
HYPV-114	HYPV-101	104,348-104,067	93	EPTV-110	90.32	MV membrane phosphoprotein, morphogenesis, essential	0.0206	140
HYPV-115	HYPV-102	104,526-104,365	53	EPTV-111	94.34	MV membrane, virulence factor, non-essential	0.0521	141
HYPV-116	HYPV-103	104,809-104,516	97	EPTV-112	85.57	Virion core protein, early stage morphogenesis	0.1194	142
HYPV-117	HYPV-104	105,935-104,793	380	EPTV-113	81.05	MV membrane, myristylated entry/fusion complex component	0.0283	143
HYPV-118	HYPV-105	106,526-105,936	196	EPTV-114	89.74	MV membrane phosphoprotein, required for morphogenesis	0.01	144
HYPV-119	HYPV-106	106,541-107,995	484	EPTV-115	82.22	DNA-dependent ATPase, DNA helicase	0.0531	145
HYPV-120	HYPV-107	108,188-107,967	73	EPTV-116	80.56	Zinc finger-like protein, involved in IV maturation to MV	0.0009	146
HYPV-121	HYPV-108	108,533-108,189	114	EPTV-117	90.35	MV membrane, entry/fusion complex component	0.0234	147
HYPV-122	HYPV-109	108,532-109,809	425	EPTV-118	76.71	DNA polymerase processivity factor, DNA replication	0.0625	148
HYPV-123	HYPV-110	109,793-110,338	181	EPTV-119	85.64	Holliday junction resolvase	0.0337	149
HYPV-124	HYPV-111	110,335-111,495	386	EPTV-120	81.35	Intermediate transcription factor large subunit (VITF-3L)	0.052	150
HYPV-125	HYPV-112	111,492-115,010	1172	EPTV-121	95.11	RNA polymerase subunit (RPO132)	0.0062	151
HYPV-126 ^#^	HYPV-113	117,881-114,996	961	EPTV-122	68.34	A-type inclusion body protein	0.0461	152
HYPV-127	HYPV-114	119,800-117,926	624	EPTV-123	71.45	A-type inclusion protein, p4c precursor	0.0434	153
HYPV-128	HYPV-115	120,206-119,856	116	EPTV-124	74.56	MV membrane fusion protein, required for MV wrapping	0.0523	154
HYPV-129	HYPV-116	120,623-120,207	138	EPTV-125	87.68	MV membrane, entry/fusion complex component	0.0243	155
HYPV-130	HYPV-117	121,539-120,637	300	EPTV-126	85.33	RNA polymerase subunit (RPO35)	0.0334	156
HYPV-131	HYPV-118	121,750-121,523	75	EPTV-127	84	MV phosphoprotein, early stage morphogenesis	0.1467	157
HYPV-132	HYPV-119	121,953-122,435	160	EPTV-128	69.81	Hypothetical protein	0.0562	159
HYPV-133	HYPV-120	123,235-122,465	256	EPTV-129	89.37	ATPase/DNA packaging protein	0.0274	160
HYPV-134	HYPV-121	123,371-123,922	183	EPTV-130	65.57	EV membrane phosphoglycoprotein, actin tail formation, C-type lectin-like domain	0.0715	161
HYPV-135	HYPV-122	123,970-124,470	166	EPTV-131	81.93	EV membrane glycoprotein, actin tail formation, C-type lectin-like domain	0.0516	162
HYPV-136	HYPV-123	124,509-125,033	174	EPTV-132	68.6	MHC class II antigen presentation inhibitor, actin tail formation	0.1094	163
HYPV-137	HYPV-124	125,073-125,918	281	EPTV-133	61.07	Concanavalin-like precursor	0.0697	
HYPV-138	HYPV-125	125,953-126,681	242	EPTV-134	54.08	EEV glycoprotein	0.0937	
HYPV-139	HYPV-126	126,724-127,554	276	EPTV-135	58.91	Hypothetical protein	0.0978	165
HYPV-140	HYPV-127	127,578-127,817	79	EPTV-136	60.26	Hypothetical protein	0.0026	
HYPV-141	HYPV-128	128,404-127,814	196	EPTV-137	63.92	CD47-like, integral membrane protein, regulation of Ca2+ influx (partial)	0.2027	167
HYPV-142	HYPV-129	128,422-128,829	135	EPTV-138	56.06	Myristoylated protein	0.266	69
HYPV-143	HYPV-130	128,826-129,587	253	EPTV-139	62.4	Hypothetical protein	0.1396	
HYPV-144	HYPV-131	130,438-129,575	287	EPTV-140	51.06	Chemokine-binding protein, interferes with chemokine-GAG interaction	0.1061	170
HYPV-145	HYPV-132	130,558-130,959	133	EPTV-141	93.98	Profilin-like protein, ATI-localized	0.0109	171
HYPV-146	HYPV-133	131,339-130,956	127	EPTV-142	63.2	Hypothetical protein	0.0876	
				EPTV-143		Hypothetical protein		
HYPV-147	-	131,608-131,895	95			Hypothetical protein		
HYPV-148	HYPV-134	131,948-133,015	355	EPTV-144	70.37	3 β-hydroxysteroid dehydrogenase/δ 5 → 4 isomerase	0.0537	174
				EPTV-145		Hypothetical protein		
HYPV-149	-	133,582-133,373	69	EPTV-146		Gasdermin homolog, pyroptosis inhibitor, immunoprevalent (partial)		177
HYPV-150	HYPV-135	133,646-134,233	195	EPTV-147	72.68	Thymidylate kinase	0.0498	178
HYPV-151	HYPV-136	134,265-135,944	559	EPTV-148	75.85	ATP-dependent DNA ligase	0.0328	180
HYPV-152	-	135,990-136,703	237	EPTV-149		BTB kelch-domain protein, NF-κB activation inhibitor (partial)		184
HYPV-153	HYPV-137	137,441-138,046	201	EPTV-150	66.67	Bcl-2 domain, blocks IFN-β promoter induction, interact with DDX3	0.0681	44
HYPV-154	-	138,094-138,441	115	EPTV-151	53.91	Hypothetical protein	0.2055	
HYPV-155	HYPV-138	138,641-139,717	358	EPTV-152	45.48	Stabilizing microtubules, negatively regulating microtubule-dependent transport	0.1061	181
HYPV-156	HYPV-139	139,781-140,428	215	EPTV-153	76.28	TLR-induced NF-κB pathway inhibitor	0.0396	182
HYPV-157	HYPV-140	140,954-140,538	138	EPTV-154	56.2	Hypothetical protein	0.141	
HYPV-158	HYPV-141	141,056-142,690	544	EPTV-155	60.45	BTB kelch-domain protein, NF-κB activation inhibitor	0.0623	184
HYPV-159	HYPV-142	142,737-143,477	246	EPTV-156	55.28	EV membrane, hemagglutinin	0.1577	185
HYPV-160	HYPV-143	143,532-144,467	311	EPTV-157	83.92	Ser/Thr protein kinase, essential for viral DNA replication	0.0248	187
HYPV-161	HYPV-144	144,505-145,470	321	EPTV-158	57.5	IL-1 receptor antagonist, virulence factor	0.1957	20
HYPV-162	HYPV-145	145,503-146,378	291	EPTV-159	53.36	KilA-N/RING finger protein (host range), E3 ubiquitin ligase, apoptosis inhibition	0.2041	21
HYPV-163	HYPV-146	146,424-147,011	195	EPTV-160	66.15	Poxin, 2′-3′-cGAMP nuclease, blocks DNA sensing and IFN induction	0.0626	188a
HYPV-164	HYPV-147	147,108-147,815	235	EPTV-161	62.39	EV type-1 membrane glycoprotein (host range), WV formation	0.0707	190
HYPV-165	HYPV-148	147,851-148,294	147	EPTV-162	59.44	Anti-apoptotic Bcl-2-like protein, inhibits NF-κB and IRF3 activation	0.0952	35
HYPV-166	-	148,323-148,751	142	MYXV-m135R	26.09	transmembrane virulence factor		
HYPV-167	-	148,753-149,505	250	EPTV-163	44.58	Z-DNA binding domain, dsRNA-binding, PKR inhibitor (host range)	0.1345	65
HYPV-168	HYPV-149	149,551-150,555	334	EPTV-164	58.68	Serpin (SPI1) (host range), apoptosis inhibitor	0.0753	208
HYPV-169	HYPV-150	150,662-151,132	156	EPTV-165	57.62	Soluble IL-1β receptor, NF-kB signal inhibitor	0.0978	200
HYPV-170	HYPV-151	151,173-152,036	287	EPTV-166	59.79	Tyrosine protein kinase-like protein	0.0752	
HYPV-171	HYPV-152	152,065-153,093	342	EPTV-167	55.82	IL-1 receptor-like protein	0.0827	201
HYPV-172	HYPV-153	153,125-155,104	659	EPTV-168	50.62	Ankyrin repeat-containing protein	0.083	
HYPV-173	-	155,154-157,172	672	EPTV-182	25.69	Ankyrin repeat-containing protein		23
HYPV-174 ^#^	HYPV-154	157,231-157,827	198	EPTV-169	47.98	Ankyrin repeat-containing protein	0.2267	
HYPV-175	-	157,902-158,273	123			Hypothetical protein		
HYPV-176	HYPV-155	158,306-159,013	235	EPTV-170	68.24	Bcl-2 domain, α-amanitin target protein, nuclear IRF3 inhibitor	0.1002	36
HYPV-177	HYPV-156	159,072-159,752	226	EPTV-171	65.33	NF-κB inhibitor, blocks CD28-mediated T cell activation	0.0709	38
HYPV-178	HYPV-157	159,796-160,020	74	EPTV-172	51.35	Endothelin precursor	0.0035	
HYPV-179	HYPV-158	160,059-160,712	217	EPTV-173	52.83	NF-κB inhibitor, blocks CD28-mediated T cell activation	0.0033	38
HYPV-180	HYPV-159	160,747-161,529	260	EPTV-174	59.46	Secreted complement-binding protein (host range)	0.0809	32
HYPV-181	-	161,564-162,049	161	SWPV-009	31.08	LAP/PHD finger-like protein		
HYPV-182	HYPV-160	162,096-162,521	141	EPTV-175	66.43	IL-18 binding protein	0.1212	
				EPTV-176		Hypothetical protein		
HYPV-183	-	162,582-162,977	131	EPTV-177	39.84	TNF receptor homolog (host range), CrmB (partial)	0.1773	2
				EPTV-178		MHC class I-like protein		
HYPV-184	-	163,038-163,241	67	EPTV-182		Ankyrin repeat-containing protein, CP77 protein (host range) (partial)		23
HYPV-185	HYPV-161	164,706-166,571	621	**EPTV-179**	41.2	Ankyrin repeat-containing protein	0.112	
				**EPTV-180**		eIF2a-like PKR inhibitor (host range)		41
				**EPTV-181**		Ankyrin repeat-containing protein		
				EPTV181.5 *		STAT1 binding, type I IFN inhibitor		24
				**EPTV-182**		Ankyrin repeat-containing protein		23
				**EPTV-183**		Soluble IL-1β receptor, NF-kB signal inhibitor		200
				**EPTV-184**		Hypothetical protein		
				**EPTV-185**		ER-localized apoptosis regulator, retains MHC I in ER		195
				**EPTV-186**		Tyrosine protein kinase-like protein		
				**EPTV-187**		TLR-induced NF-κB pathway inhibitor		
				**EPTV-188**		IL-1 receptor-like protein		
				**EPTV-189**		Bcl-2 domain, α-amanitin target protein, nuclear IRF3 inhibitor		36
				**EPTV-190**		Serpin (SPI2), CrmA, anti-apoptosis		199
				**EPTV-191**		Soluble IL-1β receptor, NF-kB signal inhibitor		200

Empty cells: no orthologs at the position. EPTV genes in the ITR regions are bolded. ^1^ If no homologous genes are found in EPTV, their closest homologs from other poxviruses are indicated. ^2^ OPG numbers according to reference [17]. ^#^ Gene starting/ending position different from original annotation. * newly identified genes in EPTV.

## Data Availability

All data necessary for the interpretation of the findings presented in this work are contained within the manuscript tables and figures.

## Abbreviations

The following abbreviations are used in this manuscript:

aa	Amino acid(s)
dsDNA	Double-stranded DNA
dN/dS	ratio of non-synonymous to synonymous nucleotide substitutions
dsRBD	dsRNA-binding domain
EFC	Entry-fusion complex
ER	Endoplasmic reticulum
ITR	Inverted terminal repeats
MV	Mature virion
OPG	Orthopoxvirus gene
ORF	Open reading frame
PKR	Protein kinase R
SCR	Short consensus repeats
TM	Transmembrane domain
TNFR	Tumor necrosis factor receptor
